# Differentiating Peanut Allergy from Sensitization in Polish Children: A Real-Life Diagnostic Model

**DOI:** 10.3390/life16030418

**Published:** 2026-03-04

**Authors:** Julia Tworowska, Aneta Krogulska

**Affiliations:** Department of Pediatrics, Allergology and Gastroenterology, Collegium Medicum in Bydgoszcz, Nicolaus Copernicus University in Torun, 87-100 Bydgoszcz, Poland; klped@cm.umk.pl

**Keywords:** peanut allergy, peanut sensitization, pediatric allergy, diagnostic prediction model, component-resolved diagnostics, basophil activation test, oral food challenge, risk stratification

## Abstract

Peanut allergy (PA) remains a major diagnostic challenge in pediatric allergy, largely due to the frequent discrepancy between immunological sensitization and clinically relevant disease. This study aimed to develop a real-life diagnostic prediction model to distinguish true peanut allergy from asymptomatic peanut sensitization in children referred for evaluation of suspected PA. In this cross-sectional study, 80 children aged 1–18 years were assessed in a tertiary allergy center in Poland. Sixty-five children with peanut sensitization underwent detailed clinical history assessment, skin prick testing, measurement of serum specific IgE including component-resolved diagnostics, basophil activation testing, and oral food challenges where clinically indicated. Clinically confirmed peanut allergy was diagnosed in 42 sensitized children. In univariate analyses, several clinical and immunological factors were associated with PA, including atopic comorbidities, peanut component sensitization, and basophil activation. Multivariate analysis identified food-induced anaphylaxis and walnut sensitization as independent factors associated with PA. In addition, a penalized diagnostic prediction model was developed to support clinical risk stratification. A multivariable diagnostic prediction model integrating clinical history and laboratory parameters demonstrated good discriminative performance in internal validation (area under the ROC curve 0.83). In conclusion, peanut allergy in sensitized children is determined by a combination of clinical and immunological factors rather than a single biomarker. Integrative diagnostic models may support risk stratification and help optimize the use of oral food challenges in specialized clinical settings, although external validation is required before broader implementation.

## 1. Introduction

Peanut allergy (PA) represents a major diagnostic challenge in pediatric allergy practice, not only because of its potential severity, but also due to the pronounced discrepancy between immunological sensitization and clinically confirmed disease [1,2]. While the prevalence of PA in children remains relatively low, sensitization to peanut is substantially more common, resulting in a large group of children with detectable peanut-specific IgE who do not develop objective allergic symptoms [1,3].

Data from the EuroPrevall population-based studies indicate that clinically confirmed peanut allergy affects approximately 1% of European children, whereas immunological sensitization to peanut is observed in about 6–9% of pediatric populations, including Central and Eastern Europe [4]. This several-fold difference creates a critical diagnostic window in which clinicians must distinguish children at genuine risk of clinical reactions from those with benign, asymptomatic sensitization. Accurate classification at this stage is essential, as both overdiagnosis and underdiagnosis may lead to significant clinical, nutritional, and psychosocial consequences [1,2].

The clinical relevance of peanut sensitization is not uniform across populations. Regional differences in exposure to aeroallergens and resulting sensitization patterns, such as birch pollen-related cross-reactivity prevalent in Central and Eastern Europe, may substantially influence the interpretation of peanut-specific IgE and component-resolved diagnostics [3,5]. Consequently, diagnostic performance of individual biomarkers and prediction models developed in other populations may not be directly transferable to Polish children, underscoring the need for population-specific approaches.

Despite advances in allergy diagnostics, distinguishing PA from asymptomatic sensitization remains challenging. Skin prick testing and measurement of peanut-specific IgE are sensitive indicators of sensitization but lack sufficient specificity to reliably identify clinically relevant disease [2,6]. Although component-resolved diagnostics and functional assays such as the basophil activation test have improved diagnostic accuracy in selected settings, no single biomarker has proven adequate as a standalone diagnostic tool across heterogeneous pediatric populations [6,7].

Oral food challenges (OFCs) remain the reference standard for confirming peanut allergy; however, their widespread use is limited by practical constraints, including substantial time and resource requirements, the need for specialized facilities and personnel, and limited acceptance by patients and caregivers [8,9]. As a result, OFCs are difficult to implement on a large scale in routine clinical practice.

Given the heterogeneity of PA, the absence of a single reliable diagnostic marker, and the limited feasibility of OFCs as a population-level tool, contemporary diagnostic strategies increasingly emphasize integrative approaches that combine clinical history with multiple immunological and functional parameters, reflecting real-life clinical decision-making [10,11,12].

The aim of this study was to develop a diagnostic prediction model to distinguish true peanut allergy from asymptomatic peanut sensitization in children referred for evaluation of suspected peanut allergy.

## 2. Materials and Methods

### 2.1. Study Design and Participants

This cross-sectional diagnostic accuracy study was conducted in a single tertiary pediatric allergy center in Poland and included 80 children aged 1–18 years referred for evaluation of suspected allergic diseases as part of routine clinical care. The study was designed as a real-life diagnostic study reflecting everyday clinical practice in a referral population.

The primary analytic study population consisted of children with peanut sensitization (peanut-specific IgE ≥ 0.35 kUA/L; *n* = 65). Within this population, the outcome of interest was clinically confirmed peanut allergy (PA), and analyses were performed comparing children with PA (*n* = 42) and peanut-sensitized but tolerant children (*n* = 23). Peanut tolerance in sensitized children was defined as either a negative oral food challenge or regular peanut ingestion without symptoms, as documented in the clinical history.

Children without peanut sensitization (*n* = 15) were included exclusively as a descriptive reference group to contextualize baseline clinical and immunological characteristics and were not used in regression analyses or model development. Accordingly, all comparative analyses and predictive modeling were performed solely within the peanut-sensitized population, with PA treated as the outcome variable and peanut-sensitized but tolerant children serving as the reference group.

**Figure 1:**
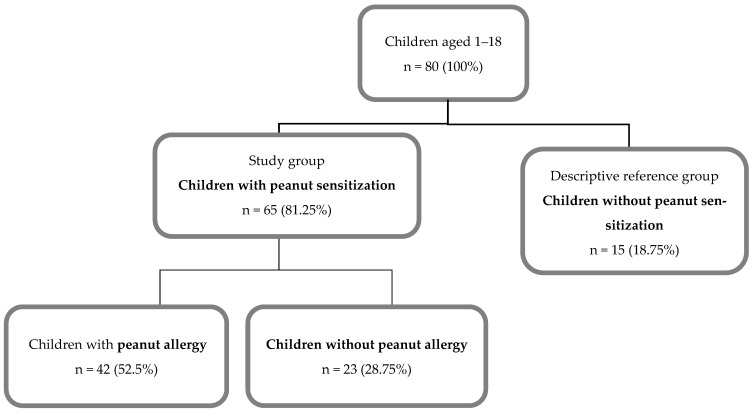
Classification of patients into study groups.

Because the non-sensitized group was not intended as an analytical comparator, a strict 1:1 matching strategy was not applied, as this would not have aligned with the diagnostic aim of the study and would have reduced statistical power. The enrollment process is illustrated in Figure 1. Baseline demographic and perinatal characteristics of the study population are summarized in Appendix A Table A1.

### 2.2. Clinical History Assessment

A detailed, structured clinical history was obtained from parents and, when appropriate, from patients aged ≥16 years, prior to diagnostic testing. The interview focused on peanut exposure history, including age at first ingestion, frequency and amount of peanut consumption, occurrence and characteristics of allergic symptoms following ingestion, timing of symptom onset, reproducibility of reactions, and potential cofactors. Additional information regarding personal and family history of atopic diseases, dietary habits, and previous allergy diagnoses was systematically recorded. This approach reflects routine clinical assessment in specialized allergy practice rather than the use of a validated questionnaire.

### 2.3. Skin Prick Tests

Skin prick tests (SPT) were performed using standardized allergen extracts (Allergopharma–Nexter, Reinbek, Germany), including peanut, common food allergens (cow’s milk, egg white and yolk, wheat, soy), and inhalant allergens (grass and tree pollens, molds, house dust mite, and animal dander), in accordance with international guidelines [11].

### 2.4. Serum Specific IgE Measurement and Component-Resolved Diagnostics

Serum specific IgE (sIgE) concentrations were assessed using a combination of multiplex and singleplex assays. Polycheck multiplex testing (Biocheck GmbH, Münster, Germany) was used exclusively for qualitative screening of sensitization profiles to selected food allergens (hazelnut, walnut, almond, cow’s milk, egg white and yolk, wheat, soy, cod) and inhalant allergens (grass and birch pollens, house dust mites, animal dander, molds) as part of routine diagnostic evaluation.

Quantitative assessment of peanut extract–specific IgE and individual peanut components was performed using fluorescence enzyme immunoassay (FEIA; ImmunoCAP 100, Thermo Fisher Scientific, Waltham, MA, USA), which served as the basis for model development and interpretation. Specific IgE to peanut extract and individual peanut components (Ara h 1, Ara h 2, Ara h 3, Ara h 6, Ara h 8, and Ara h 9) was measured using ImmunoCAP FEIA. Sensitization to peanut extract and individual components was defined as sIgE ≥ 0.1 kUA/L.

### 2.5. Basophil Activation Test

Basophil activation testing (BAT) was performed using the FlowCAST assay (BÜHLMANN Laboratories AG, Schönenbuch, Switzerland) on whole blood collected in K2EDTA tubes and analyzed on a FACSCalibur flow cytometer (Becton Dickinson, Franklin Lakes, NJ, USA). Basophil activation was quantified as the percentage of CD63-positive basophils in response to peanut extract, with anti-FcεRI antibody and fMLP used as positive controls. Standardized reagents provided with the assay were used according to the manufacturer’s instructions. A positive BAT result was defined as >15% CD63-positive basophils. In samples with spontaneous activation >2%, a stimulation index (stimulated/unstimulated) ≥2 was required for positivity, according to manufacturer recommendations [13].

### 2.6. Oral Food Challenge

In children without reliable prior exposure history, clinical classification relied on oral food challenge (OFC) results, or on a combination of convincing recent reactions and supportive sensitization/functional testing profiles when OFC was ethically or practically not feasible.

OFCs were performed after completion of diagnostic testing in accordance with AAAAI guidelines [8,9]. Peanut powder (44 g protein/100 g) was administered mixed with a tolerated fruit puree or dairy dessert in nine incremental doses at 15 min intervals, to a cumulative dose of 8 g peanut protein. Stopping criteria followed guideline recommendations, and anaphylaxis was defined according to EAACI criteria [12].

OFC was not performed in children with a convincing history of peanut-induced reactions within the preceding 12 months combined with Ara h 2 > 1.0 kUA/L (*n* = 12), or in children who regularly consumed peanut without symptoms (*n* = 4). In accordance with routine clinical practice, OFC was not performed in children with recent convincing reactions supported by sensitization profiles or in children with regular asymptomatic peanut ingestion; this may have introduced classification bias.

### 2.7. Statistical Analysis

Statistical analyses were conducted using Statistica 13.3 (StatSoft, Krakow, Poland). Data distribution was assessed using the Shapiro–Wilk test, and the choice of parametric or non-parametric statistical tests was based on these results. Categorical variables are presented as absolute numbers and percentages, while continuous variables are reported as means (SD) for normally distributed data or medians (IQR) for non-normally distributed data. Group comparisons were performed using Student’s *t*-test or the Mann–Whitney U test for continuous variables, as appropriate, and the chi-square test for categorical variables. Statistical significance was set at *p* < 0.05.

Univariate logistic regression was used to screen candidate predictors associated with peanut allergy (PA). Multivariable model development was primarily performed using penalized logistic regression with LASSO regularization, with the penalty parameter (λ) tuned using cross-validation. Predictors selected by LASSO were subsequently entered into a standard multivariable logistic regression model to obtain regression coefficients (B), odds ratios (OR), and 95% confidence intervals (CI).

As a sensitivity analysis, a multivariable logistic regression model with forward stepwise selection (entry criterion *p* = 0.05, removal criterion *p* = 0.10) was also performed. Model fit was evaluated using Cox–Snell and Nagelkerke R^2^. Because variable selection was performed using penalized regression, *p*-values and confidence intervals should be interpreted cautiously, as they are derived from post-selection estimation and do not follow the assumptions of classical maximum likelihood inference.

Discriminative performance was assessed using receiver operating characteristic (ROC) curve analysis, and area under the ROC curve (AUC) was used as the primary performance measure. Internal validation was performed within the cross-validation procedure used for LASSO tuning.

In the primary analytical population, 42 children were classified as having peanut allergy (events) and 23 as peanut-sensitized but tolerant (non-events). Candidate predictors were selected based on clinical relevance and results of univariate screening and entered into penalized logistic regression with LASSO regularization. Following LASSO-based variable selection, nine predictors were retained in the final refitted multivariable logistic regression model. The resulting events-per-variable (EPV) ratio in the final model was approximately 4.7 (42 events/9 predictors). Given the modest EPV and the potential risk of overfitting, penalized regression with cross-validation-based tuning of the penalty parameter (λ) was applied as a shrinkage strategy to improve model stability.

### 2.8. Ethics

This study was approved by the local Bioethics Committee (No. KB 234/2019). Written informed consent was obtained from all parents or legal guardians and from patients aged 16 years or older.

## 3. Results

### 3.1. Study Population

A total of 80 children aged 1–18 years were included in the study (51% male). Among the 65 peanut-sensitized children constituting the primary analytical population, 42 (64.6%) met criteria for clinically confirmed peanut allergy (PA), while 23 (35.4%) were classified as peanut-sensitized but tolerant. Fifteen non-sensitized children were included as a descriptive reference group.

### 3.2. Clinical History and Peanut Exposure

A history of prior peanut ingestion was reported in 40 children (50% of the total population). Among these, 26 children (65%) developed clear symptoms consistent with PA, 3 (7.5%) reported ambiguous symptoms, and 11 (27.5%) remained asymptomatic. In the remaining 40 children (50%), peanut ingestion history was unknown or could not be reliably assessed.

In children diagnosed with PA, the median age at symptom onset was 2.5 years (range: 8 months–5 years). Cutaneous manifestations, including urticaria, erythema, and pruritus, were observed in 25 symptomatic children (96.5%). Respiratory symptoms (mainly cough and wheezing) occurred in 17 cases (65.4%), behavioral symptoms in 15 (57.7%), and gastrointestinal symptoms in 12 (46.2%). Syncope was reported in 6 children (23.1% of PA cases).

### 3.3. Immunological and Functional Test Results

Within the peanut-sensitized population, children with PA demonstrated significantly higher sensitization to walnut and to peanut components Ara h 1, Ara h 2, and Ara h 6 compared with children without peanut allergy. Additionally, SPT wheal size to peanut was significantly larger in the PA group (all *p* < 0.05). No statistically significant differences were observed for other food or inhalant allergens.

Basophil activation in response to peanut extract was markedly higher in children with PA compared with children without PA (40.3 ± 30.8% vs. 9.1 ± 14.2%, *p* < 0.05). Key peanut-related immunological parameters are summarized in Table 1, while detailed sensitization profiles to additional food and inhalant allergens are provided in Appendix A Table A2.

### 3.4. Oral Food Challenge Outcomes

Open oral food challenges were performed in 49 children (61.3% of the total population). A negative OFC was observed in 27 children (55%), while 22 children (45%) exhibited objective allergic reactions.

The most frequently reported subjective symptoms during OFC included pruritus (61.3%), anxiety or behavioral changes (77.3%), throat itching (72.7%), and abdominal pain (22.7%). Objective clinical findings comprised urticaria (90.9%), angioedema (68.2%), rhinorrhea (54.5%), cough (50%), vomiting (40.9%), wheezing (31.8%), diarrhea (27.3%), stridor (13.6%), and hypotension (13.6%).

Anaphylaxis was diagnosed in 18 of 22 positive OFCs (81.8%), most commonly of grade III severity (44%). The mean tolerated cumulative dose was 902.5 ± 1385.1 mg peanut protein, and the mean eliciting dose was 856.0 ± 511.2 mg.

### 3.5. Univariate and Multivariate Regression Analyses

**Figure 2:**
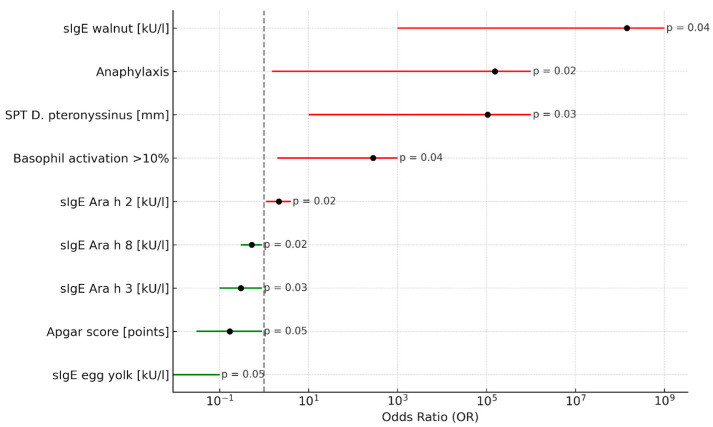
Univariate logistic regression analysis of factors associated with peanut allergy in peanut-sensitized children.

In univariate logistic regression analysis (Model I), factors significantly associated with PA included atopic dermatitis (OR = 13.00; 95% CI: 3.42–49.42; *p* < 0.0001), asthma (OR = 5.00; 95% CI: 1.86–13.47; *p* = 0.002), food-induced anaphylaxis (OR = 16.87; 95% CI: 5.65–50.35; *p* < 0.0001), cow’s milk allergy (OR = 3.43; 95% CI: 1.37–8.60; *p* = 0.004), tree nut allergy (OR = 2.00; 95% CI: 0.76–5.23; *p* = 0.005), walnut sensitization (OR = 6.51; 95% CI: 2.75–18.17; *p* < 0.0001), peanut SPT wheal size, sIgE to peanut extract, sIgE to Ara h 1, Ara h 2, and Ara h 6, as well as a positive BAT response to peanut extract (all *p* < 0.05). Full results are summarized in Table 2. The strength and direction of associations identified in univariate analyses are summarized in Figure 2.

In multivariate analysis (Model II), only food-induced anaphylaxis (aOR = 19.33; 95% CI: 7.41–60.62; *p* < 0.0001) and walnut sensitization (aOR = 4.67; 95% CI: 2.33–15.40; *p* = 0.016) remained independently associated with PA (Table 2).

### 3.6. Predictive Model for Peanut Allergy

A multivariable diagnostic prediction model for PA was developed using penalized logistic regression with LASSO regularization. Predictors selected by LASSO were subsequently refitted using standard multivariable logistic regression to obtain regression coefficients (B), ORs and 95% CIs (Table 3). As a sensitivity analysis, a forward stepwise logistic regression model was also evaluated. The final model included nine variables selected by penalized regression; five variables showed positive regression coefficients and four showed negative coefficients in the refitted multivariable model (Table 3). The sign of the coefficients reflects the direction of association within the predictive model and should not be interpreted as independent causal effects. The magnitude of some odds ratios reflects post-selection instability related to penalized regression, correlation between predictors, and limited sample size, rather than true biological effect sizes.

**Figure A1:**
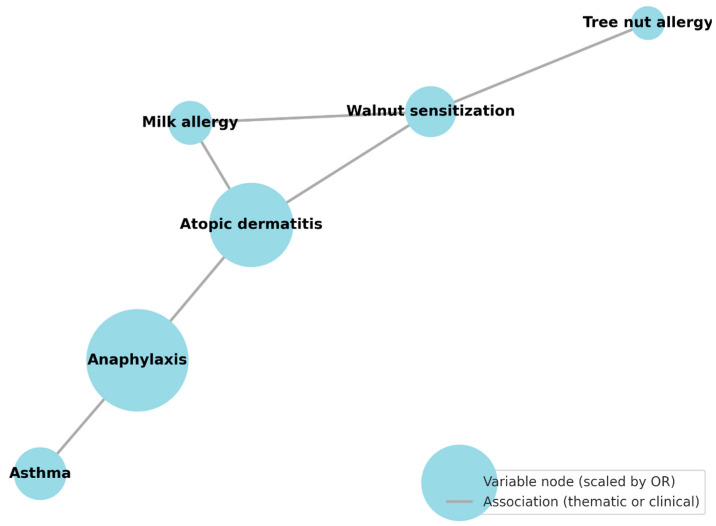
A network-based visualization of the predictive model.

The model showed high apparent classification accuracy in the derivation dataset (96.3% at the selected probability threshold). Discrimination assessed by ROC analysis yielded an AUC of 0.83. Model fit of the refitted multivariable logistic regression model was confirmed by a Cox–Snell R^2^ of 0.693 and a Nagelkerke R^2^ of 0.925. A graphical representation of the model structure and the network-based prognostic map is presented in Figure A1 in Appendix A.

Model discrimination was consistent in internal validation performed within the cross-validation procedure used for LASSO tuning, supporting the ability of the model to differentiate clinical phenotypes within a real-life population.

## 4. Discussion

In this real-life diagnostic study of peanut-sensitized children, we identified a set of clinical and immunological factors associated with clinically confirmed peanut allergy and developed a multivariable prediction model to support risk stratification in routine pediatric allergy practice. Our findings confirm the diagnostic complexity inherent in distinguishing true peanut allergy from asymptomatic sensitization and highlight the value of integrative approaches combining clinical history with multiple laboratory parameters. Importantly, a clear distinction should be made between analyses aimed at identifying factors associated with peanut allergy and the development of a diagnostic prediction model optimized for clinical risk stratification.

In univariate analyses, several established risk factors for peanut allergy, including atopic dermatitis, asthma, food-induced anaphylaxis, and coexisting food allergies, were strongly associated with peanut allergy [14,15,16,17]. Some variables retained in the penalized predictive model, such as Apgar score, egg yolk sensitization, or SPT to *Dermatophagoides pteronyssinus*, may reflect broader atopic or immunological patterns rather than direct causal relationships with peanut allergy. Their inclusion likely results from complex correlation structures captured by penalized regression models optimized for prediction rather than etiological inference and should not be interpreted as independent biological predictors.

These findings are consistent with previous studies demonstrating that peanut allergy often occurs within a broader atopic phenotype and is associated with more severe allergic manifestations. However, when variables were analyzed simultaneously in a standard multivariable model, only a history of food-induced anaphylaxis and walnut sensitization remained independently associated with peanut allergy. This underscores the importance of considering interactions between clinical and immunological factors rather than relying on isolated predictors.

The very large odds ratios observed for some predictors likely reflect post-selection instability related to limited sample size and correlations between clinical and immunological variables rather than true effect magnitude; therefore, effect sizes should be interpreted cautiously.

A history of food-induced anaphylaxis emerged as the strongest independent factor associated with peanut allergy. Rather than implying causality, this finding reflects a more severe atopic endotype. From a diagnostic perspective, it highlights the continued importance of detailed clinical history in peanut-sensitized children, in line with current EAACI recommendations [2,12].

Walnut sensitization was also independently associated with peanut allergy and may represent a surrogate marker of broader immune reactivity, shared storage protein sensitization, or cumulative allergen exposure, all of which have been linked to clinically relevant food allergy [5,18]. Importantly, this association may be influenced by regional sensitization patterns, underscoring the relevance of population-specific analyses.

Consistent with previous reports, sensitization to peanut components Ara h 1, Ara h 2, and Ara h 6, as well as basophil activation in response to peanut extract, was more pronounced in allergic compared with tolerant children [3,7,18]. However, none of these markers retained independent significance in multivariable modeling, reinforcing the multifactorial nature of peanut allergy and supporting the concept that no single biomarker is sufficient for reliable diagnosis across diverse pediatric populations. Large-scale data from Poland indicate that peanut is among the most prevalent food allergens in children; however, most available studies focus on molecular sensitization profiles without systematic clinical correlation, limiting their utility in distinguishing true peanut allergy from asymptomatic sensitization [19]. These observations are consistent with findings from a recent Polish proof-of-concept study, which demonstrated that component-resolved diagnostics, although associated with oral food challenge outcomes, lacked sufficient accuracy to reliably replace challenge-based diagnosis in peanut-sensitized children [20].

Building on these observations, we developed a multivariable diagnostic prediction model integrating nine clinical and laboratory variables. The model demonstrated high classification accuracy in the derivation population. Direct numerical comparison with prediction models derived from population-based prevention trials, such as the LEAP study, was not appropriate due to fundamental differences in study design, target population, and clinical objectives. Importantly, the model was developed in a real-life referral population enriched for peanut-sensitized children, reflecting the diagnostic uncertainty commonly encountered in tertiary care. In this context, the model is not intended to replace oral food challenges but to assist in stratifying risk and optimizing the use of resource-intensive diagnostic procedures [2,8,10].

### Strengths and Limitations

The major strength of this study lies in its real-life setting and multimodal diagnostic design, consistent with current EAACI guidance [11,12]. However, several limitations should be acknowledged.

First, no formal sample size or statistical power calculation was performed, as the study was exploratory in nature and based on a real-life referral population. Consequently, the modest sample size may limit the stability of multivariable and penalized regression estimates and the single-center nature and modest sample size may limit generalizability.

Second, oral food challenges were not performed in all children, due to ethical and practical considerations, potentially introducing classification bias. Although double-blind, placebo-controlled oral food challenges (DBPCFC) are considered the reference standard for food allergy diagnosis in research settings, their routine use in real-life pediatric allergy practice is limited; therefore, diagnostic classification in this study followed established real-life diagnostic pathways combining open oral food challenges, clinical history, and supportive immunological testing, in line with current EAACI recommendations [11,12]. This approach reflects routine clinical decision-making in tertiary allergy centers rather than a controlled diagnostic accuracy study. Third, external validation was not feasible at this stage due to limited sample size.

Although the predictive model demonstrated promising performance in our population, its broader clinical use may be constrained by the availability of certain diagnostic tools. In particular, component-resolved diagnostics (CRD) and the basophil activation test (BAT) are both required to compute the model. These advanced assays, though increasingly used in tertiary settings, remain largely unavailable in many primary or regional centers due to financial, logistic, or reimbursement limitations. In addition, the study population represents a referral-enriched, high-risk population, which further limits the generalizability of the model to similar tertiary allergy care settings. Therefore, implementation may be restricted to specialized allergy units with appropriate infrastructure. Given that the study population consisted of children referred to a tertiary allergy center, the model is most applicable to high-risk, peanut-sensitized populations rather than unselected community populations.

It is well recognized that a predictive model may perform well in its development population yet fail to generalize to new patient populations. In the present study, external validation was not possible; however, we plan to address this by collecting new data in an independent population. Meanwhile, internal validation was conducted using ROC analysis and calculation of the area under the curve (AUC), yielding an AUC of 0.83, which provides a preliminary estimate of the model’s discriminative ability. Formal assessment of model calibration was not performed, as calibration plots would be of limited interpretability in the context of the modest sample size and the absence of external validation. Although encouraging, further evaluation in external datasets is essential to confirm reproducibility and clinical utility.

## 5. Conclusions

In conclusion, this real-life study demonstrates that peanut allergy in sensitized children is associated with a combination of clinical and immunological factors rather than a single diagnostic marker. By integrating routinely available clinical history and laboratory data, we developed a diagnostic prediction model that may support risk stratification in peanut-sensitized children in specialized clinical settings. While the model shows promising performance, external validation is required before routine clinical use. These findings reinforce the need for population-specific, integrative diagnostic approaches to improve the management of peanut allergy and reduce unnecessary diagnostic procedures.

## Figures and Tables

**Table 1:** Key immunological parameters in peanut-sensitized children with and without peanut allergy.

Parameter	Children Without PA, n = 23 (%)	Children with PA, n = 42 (%)	*p*
sIgE (CRD) mean ± SD [kUA/L]			
peanut extract	7.55 ± 19.38	27.29 ± 30.72	<0.05
Ara h 1	0.64 ± 1.64	11.13 ± 26.84	<0.05
sIgE Ara h 2	2.65 ± 11.27	21.08 ± 30.41	<0.05
sIgE Ara h 3	0.56 ± 1.54	5.34 ± 16.04	0.07
sIgE Ara h 6	2.27 ± 9.98	16.93 ± 28.13	<0.05
sIgE Ara h 8	18.66 ± 30.85	3.13 ± 4.97	0.39
sIgE Ara h 9	2.25 ± 9.04	4.31 ± 16.43	0.35
SPT mean ± SD [mm]			
Peanut	3.46 ± 2.06	4.95 ± 2.33	0.01
BAT mean ± SD [%]			
BAT 0	11.26 ± 18.51	7.77 ± 14.45	0.93
BAT Fc epsilon RI	54.10 ± 30.51	64.30 ± 28.33	0.07
BAT f-MLP	33.63 ± 25.51	31.27 ± 20.53	0.85
BAT peanut extract	9.12 ± 14.15	40.30 ± 30.84	<0.05

**Table 2:** Logistic regression analysis of risk factors associated with peanut allergy in the studied children.

Variable	Model I OR (95% CI)	*p*	Model II aOR (95% CI)	*p*
Atopic dermatitis	13.00 (3.42–49.42)	<0.0001		
Asthma	5.00 (1.86–13.47)	0.002		
Anaphylaxis	16.87 (5.65–50.35)	<0.0001	19.33 (7.41–60.62)	<0.0001
Milk allergy	3.43 (1.37–8.60)	0.004		
Tree nut allergy	2.00 (0.76–5.23)	0.005		
Walnut sensitization	6.51 (2.75–18.17)	<0.0001	4.67 (2.33–15.40)	0.016

**Table 3:** Multivariate predictive model of peanut allergy. Variables were selected using LASSO penalized logistic regression and refitted in a standard multivariable model; coefficients represent post-selection estimates.

Variable	B	OR	*p*
Apgar score [points]	−1.79	0.17	0.05
Anaphylaxis	11.95	154,571.72	0.02
sIgE Ara h 2 [kU/L]	0.76	2.14	0.02
sIgE Ara h 3 [kU/L]	−1.20	0.30	0.03
sIgE Ara h 8 [kU/L]	−0.63	0.53	0.02
Basophil activation >15%	5.64	281.32	0.04
sIgE walnut [kU/L]	18.78	142,944,280.13	0.04
sIgE egg yolk [kU/L]	−16.58	<0.01	0.05
SPT D. pteronyssinus [mm]	11.58	106,765.39	0.03

**Figure A2:**
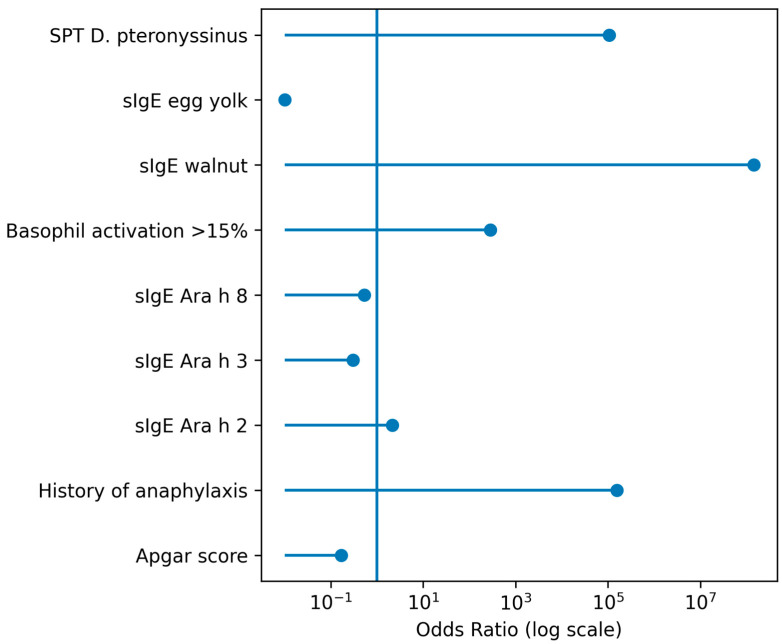
Forest plot of the multivariable predictive model for peanut allergy. Odds ratios (ORs) are displayed on a logarithmic scale. Horizontal lines indicate the direction and relative magnitude of regression coefficients within the predictive model. The vertical reference line represents OR = 1. Coefficients represent post-selection estimates following LASSO regularization and should not be interpreted as independent causal effects.

## Data Availability

The data presented in this study are not publicly available due to ethical and privacy restrictions related to pediatric patient data. Anonymized datasets may be made available from the corresponding author upon reasonable request and with approval from the local Bioethics Committee.

## Abbreviations

The following abbreviations are used in this manuscript:

AUC	Area under the curve
BAT	Basophil activation test
CI	Confidence interval
CRD	Component-resolved diagnostics
EAACI	European Academy of Allergy and Clinical Immunology
FEIA	Fluorescence enzyme immunoassay
fMLP	N-formylmethionyl-leucyl-phenylalanine
IgE	Immunoglobulin E
IQR	Interquartile range
kUA/L	kilo units of allergen-specific IgE per liter
OFC	Oral food challenge
OR	Odds ratio
PA	Peanut allergy
ROC	Receiver operating characteristic
SD	Standard deviation
sIgE	Specific immunoglobulin E
SPT	Skin prick test

## Appendix A

This Appendix A provides additional descriptive data and extended visualizations that complement the main analyses while preserving the clarity and focus of the primary manuscript.

Table A1 presents baseline demographic and clinical characteristics of children with peanut sensitization compared with non-sensitized control children. This table is included to provide contextual background and to allow comparison of general population characteristics between sensitized and non-sensitized participants. These data were not used as comparators in regression analyses or model development and are reported for descriptive purposes only.

**Table A1:** Baseline characteristics of the peanut-sensitized population and the descriptive non-sensitized reference group.

Parameter	Children with Peanut Sensitization *n* = 65 (100%)	Children Without Peanut Sensitization *n* = 15 (100%)
Mean age ± SD (years)	5.74 ± 3.21	4.07 ± 2.28
Min–max (years)	1–15	1–10
Sex, *n* (%)		
Female	16 (24.61)	7 (46.67)
male	49 (75.39)	8 (53.33)
Type of delivery, *n* (%)		
Vaginal	41 (63.08)	8 (53.33)
cesarean	24 (36.92)	7 (46.67)
Gestational age, weeks (*n* %)		
<37	4 (6.16)	1 (6.67)
38–40	50 (76.92)	12 (80)
>40	11 (16.92)	2 (13.33)
Apgar score, points (*n* %)		
<8	3 (4.61)	1 (6.67)
8–10	62 (95.39)	14 (93.33)
Birth weight [g] (mean ± SD)	3374.85 ± 550.12	3455 ± 319.57
Place of residence, *n* (%)		
Rural	19 (29.23)	6 (39.9)
urban	46 (70.77)	9 (60.1)
Atopy, *n* (%)		
1 parent	30 (46.15)	4 (26.66)
both parents	12 (18.46)	2 (13.33)
siblings	18 (27.69)	3 (20)

Table A2 summarizes detailed sensitization profiles to additional food and inhalant allergens assessed by serum specific IgE (sIgE) measurements and skin prick testing (SPT). These results reflect the broader atopic background of the study population and were collected as part of routine diagnostic evaluation. Unless explicitly stated in the Methods section, these variables were not included in the final predictive model and are provided to enhance transparency and reproducibility.

**Table A2:** Food and inhalant sensitization—sIgE, SPT, and BAT in children with sensitization and allergy to peanuts.

Parameter	Children Without PA, *n* = 23 (%)	Children with PA, *n* = 42 (%)	*p*
sIgE mean ± SD [kUA/L]			
Hazelnut	27.34 ± 42.03	24.71 ± 36.96	0.77
Walnut	4.53 ± 20.35	8.97 ± 23.04	0.01
sIgE almond	11.69 ± 28.54	10.31 ± 24.90	0.80
sIgE cow’s milk	14.66 ± 33.30	19.63 ± 38.44	0.88
sIgE white egg	10.33 ± 25.74	20.01 ± 84.10	1.0
sIgE egg yolk	4.51 ± 20.35	1.34 ± 4.68	0.19
sIgE cod	5.38 ± 20.94	3.10 ± 16.03	0.36
sIgE soya	4.44 ± 15.88	5.64 ± 16.38	0.71
sIgE wheat	6.10 ± 20.97	3.87 ± 13.45	0.53
sIgE birch pollen	33.37 ± 43.69	38.41 ± 57.33	0.81
sIgE grass pollen	16.55 ± 34.61	15.05 ± 41.18	0.62
sIgE D. pteronysinus	21.29 ± 41.11	37.96 ± 66.13	0.19
sIgE D. farinea	17.33 ± 35.55	30.77 ± 60.01	0.47
sIgE dog dander	9.09 ± 28.03	17.43 ± 32.93	0.60
sIgE cat dander	10.78 ± 26.42	12.12 ± 30.61	0.37
sIgE Alternaria alternata	1.14 ± 3.88	1.99 ± 9.53	0.71
SPT mean ± SD [mm]			
SPT cow’s milk	3.17 ± 4.35	3.73 ± 4.42	0.59
SPT white egg	4.00 ± 4.03	2.80 ± 3.81	0.21
SPT egg yolk	0.92 ± 1.69	0.85 ± 1.56	0.95
SPT cod	0.29 ± 1.08	0.37 ± 1.73	0.62
SPT soya	1.33 ± 2.16	1.83 ± 2.48	0.42
SPT wheat	0.71 ± 1.55	1.12 ± 1.76	0.31
SPT birch pollen	5.25 ± 3.65	5.07 ± 4.55	0.59
SPT grass pollen	2.83 ± 3.36	2.90 ± 3.53	0.96
SPT D. pteronysinus	2.88 ± 4.31	5.51 ± 10.19	0.19
SPT D. farinea	3.75 ± 4.10	4.05 ± 4.52	0.59
SPT dog dander	2.33 ± 3.33	3.2 ± 3.78	0.37
SPT cat dander	2.29 ± 3.58	1.88 ± 3.47	0.54
SPT Alternaria alternata	0.96 ± 2.16	1.05 ± 2.25	0.91

Figure A1 presents an extended, network-based visualization of the diagnostic prediction model. This figure provides a graphical representation of the relationships between clinical and immunological variables selected by penalized regression and their contribution to the final model. The figure is intended as an illustrative aid to facilitate interpretation of the multivariable structure of the model rather than as a standalone analytical result.

**Appendix A Figure A2**

The materials included in this Appendix A do not alter the main conclusions of the study but provide complementary visualizations of the predictive model. Figure A1 illustrates the multivariable structure and interrelationships between predictors, whereas Figure A2 provides a conventional statistical representation of coefficient direction and magnitude, thereby contextualizing the univariate findings presented in the main text.
